# Growth of the facultative chemolithoautotroph *Ralstonia eutropha* on organic waste materials: growth characteristics, redox regulation and hydrogenase activity

**DOI:** 10.1186/s12934-019-1251-5

**Published:** 2019-11-18

**Authors:** Anna Poladyan, Syuzanna Blbulyan, Mayramik Sahakyan, Oliver Lenz, Armen Trchounian

**Affiliations:** 1 Department of Biochemistry, Microbiology and Biotechnology, Biology Faculty, Yerevan State University, 1 A. Manoukian Str, 0025 Yerevan, Armenia; 2 Research Institute of Biology, Biology Faculty, Yerevan State University, 1 A. Manoukian Str, 0025 Yerevan, Armenia; 3 Institute of Chemistry, Technical University of Berlin, 17. Juni 135, 10623 Berlin, Germany

**Keywords:** *Ralstonia eutropha* H16, Organic wastes, Hydrogenase activity, Redox stress, FoF_1_-ATPase

## Abstract

**Background:**

The chemolithoautotrophic β-proteobacterium *Ralstonia eutropha* H16 (*Cupriavidus necator*) is one of the most studied model organisms for growth on H_2_ and CO_2_. *R. eutropha* H16 is also a biologically significant bacterium capable of synthesizing O_2_-tolerant [NiFe]-hydrogenases (Hyds), which can be used as anode biocatalysts in enzyme fuel cells. For heterotrophic growth of *R. eutropha*, various sources of organic carbon and energy can be used.

**Results:**

Growth, bioenergetic properties, and oxidation–reduction potential (ORP) kinetics were investigated during cultivation of *R. eutropha* H16 on fructose and glycerol or lignocellulose-containing brewery spent grain hydrolysate (BSGH). BSGH was used as carbon and energy source by *R. eutropha* H16, and the activities of the membrane-bound hydrogenase (MBH) and cytoplasmic, soluble hydrogenase (SH) were measured in different growth phases. Growth of *R. eutropha* H16 on optimized BSGH medium yielded ~ 0.7 g cell dry weight L^−1^ with 3.50 ± 0.02 (SH) and 2.3 ± 0.03 (MBH) U (mg protein)^−1^ activities. Upon growth on fructose and glycerol, a pH drop from 7.0 to 6.7 and a concomitant decrease of ORP was observed. During growth on BSGH, in contrast, the pH and ORP stayed constant. The growth rate was slightly stimulated through addition of 1 mM K_3_[Fe(CN)_6_], whereas temporarily reduced growth was observed upon addition of 3 mM dithiothreitol. The overall and *N*,*N*′-dicyclohexylcarbodiimide-sensitive ATPase activities of membrane vesicles were ~ 4- and ~ 2.5-fold lower, respectively, upon growth on fructose and glycerol (FGN) compared with only fructose utilization (FN). Compared to FN, ORP was lower upon bacterial growth on FGN, GFN, and BSGH.

**Conclusions:**

Our results suggest that reductive conditions and low ATPase activity might be signals for energy depletion, which, in turn, leads to increased hydrogenase biosynthesis to overcome this unfavorable situation. Addition of fructose or microelements have no, or a negative, influence on hydrogenase activity. Organic wastes (glycerol, BSGH) are promising carbon and energy sources for the formation of biomass harboring significant amounts of the biotechnologically relevant hydrogenases MBH and SH. The results are valuable for using microbial cells as producers of hydrogenase enzymes as catalysts in enzymatic fuel cells.

## Background

The facultative chemolithoautotrophic β-proteobacterium *Ralstonia eutropha* H16 is a biotechnologically relevant bacterium capable of synthesizing a range of metabolites and bioplastics both heterotrophically from organic substances and lithoautotrophically [1–5]. For lithoautotrophic growth on H_2_, CO_2_ and O_2_, *R. eutropha* H16 employs O_2_-tolerent [NiFe]-hydrogenases (Hyds), one of which has been applied as an anode biocatalyst in enzymatic fuel cells [6, 7]. *R. eutropha* H16 possesses four different O_2_-tolerant [NiFe]-Hyds involved in H_2_ turnover: a membrane-bound Hyd (MBH), a cytoplasmic soluble Hyd (SH), an actinobacterial-type Hyd (AH) and a regulatory Hyd (RH) [8–16].

Genes for MBH and SH, which are the energy-conserving hydrogenases of *R. eutropha* H16, are located in two operons: a MBH operon (21-kb) and a SH operon (10-kb), separated by 59-kb [5, 12, 14]. A number of structural and accessory proteins are involved in the complex biosynthesis of all four hydrogenases. The MBH comprises a Ni–Fe active site-containing large subunit (HoxG), a Fe–S cluster-accommodating electron-transferring small subunit (HoxK), and a membrane-spanning cytochrome *b* (HoxZ) [9, 12, 16]. The periplasmically oriented enzyme is involved in H_2_-driven respiration with O_2_ as the terminal electron acceptor. SH is a cytoplasmic enzyme that directly reduces NAD^+^ to NADH at the expense of H_2_, and thus generates reducing equivalents [12, 14, 15, 17]. The NADH:quinone oxidoreductase oxidizes some of the NADH produced by SH and the released electrons are transported through the respiratory chain to generate a proton gradient (Δµ_H+_), which is eventually used for ATP synthesis. The hetero-hexameric SH is composed of the subunits HoxHYFUI_2_ and harbors multiple redox cofactors: a [Ni–Fe] active site, an electron relay of iron–sulfur clusters, and two non-covalently bound flavin mononucleotides (FMN) [12, 17]. The RH controls hydrogenase gene transcription according to the availability of H_2_, while the AH belongs to the sub-class of high-affinity hydrogenases, which are thought to be connected to the respiratory chain [8, 11, 12].

Different organic carbon and energy sources can be used for heterotrophic growth of *R. eutropha* H16, including intermediates of the tricarboxylic acid (TCA) cycle, sugar acids, fatty acids, amino acids, alcohols, and aromatic compounds [2–5]. Under certain conditions, *R. eutropha* H16 accumulates biodegradable plastics in the form of polyhydroxyalkanoates (PHA) in granules which can make up to 80% of the cellular dry weight (CDW). PHAs serve as storage compounds for carbon and energy [5, 18, 19]. In addition, relatively cheap carbon sources, e.g. organic wastes, can also be used to cultivate *R. eutropha* H16 [3, 14, 18, 19]. Among them is glycerol, which is widely recognized as an industrially important waste product. Glycerol is a by-product of biomass, biodiesel, vegetable oils, and animal fats production [20]. Another attractive waste material for bacterial growth is lignocellulosic biomass, such as brewery-spent grains (BSG) [21, 22]. Beer is consumed in large quantities all over the world, and the estimated annual production of BSG worldwide is approximately 38.6 × 10^6^ tons [21–24]. BSG hydrolysate (BSGH) is typically a hemicellulosic hydrolysate, which can contain pentose and hexose sugars, formic acid, acetic acid, aromatic compounds, as well as (micro) elements, such as, sodium, potassium, calcium, magnesium, iron, manganese, copper, zinc, aluminum, barium, strontium, phosphorus, sulfur, chromium and silicon [22, 25, 26]. Some of these substrates can be utilized by *R. eutropha* H16.

Although heterotrophic growth of *R. eutropha* H16 is best on several organic substrates, fructose and glycerol are more widely used, as they guarantee optimal conditions for Hyd enzyme synthesis [3, 14]. Fructose is mainly transported into the cell by an ABC-type transporter [5] and catabolized through the Entner–Doudoroff pathway. Glycerol supports very slow growth of *R. eutropha* H16, which, however, leads to strong expression of genes for Hyds and enzymes of the Calvin–Benson–Bassham (CBB) cycle, the key components of lithoautotrophic metabolism [1, 18, 19]. Glycerol is probably transported into the cell via facilitated diffusion mediated by the glycerol uptake facilitator protein GlpF [3]. Two proteins, a glycerol kinase and a glycerol-3-phosphate dehydrogenase, are involved in the phosphorylation of intracellular glycerol to glycerol 3-phosphate and the subsequent conversion into dihydroxyacetone phosphate. The latter is either introduced into gluconeogenesis or catabolized through the Entner–Doudoroff pathway to pyruvate and then to acetyl-CoA.

Oxidation–reduction processes are indispensable for bacterial growth and the adaptation of microorganisms to different environmental conditions [27–29]. Consequently, the oxidation–reduction (redox) potential (ORP) depends strongly on the metabolic state and, thus, also affects end product formation. In previous work, a decrease of ORP was observed in parallel with a decrease of the extracellular pH suggesting a direct relationship between the ORP and Δµ_H+_ [28, 30]. Moreover, a role of the H^+^-pumping F_O_F_1_-ATP synthase, an enzyme of bioenergetic relevance, which is responsible for maintaining of Δµ_H+_ and for ATP synthesis under certain conditions in redox sensing by bacteria, has also been suggested [27, 28, 30]. However, the mechanisms of redox sensing and the influence of ORP on bacterial metabolism are still barely understood for most bacterial species.

In the present work, we investigated the growth properties of *R. eutropha* H16 on different carbon sources (fructose and glycerol, BSGH) and analyzed the corresponding kinetic changes of ORP and pH. We showed for the first time that cells grown on BSGH show MBH and SH activity. The addition of a reducing compound to a growing culture led to a transient growth suppression, but growth recovered a couple of hours after addition. Moreover, bioenergetic properties, such as intracellular pH and ATPase activity and its inhibition by *N*,*N*′-dicyclohexylcarbodiimide (DCCD) were investigated under different conditions.

## Materials and methods

### Growth media and cultivation conditions of bacteria

*Ralstonia eutropha* H16 bacteria were grown heterotrophically in Fructose–Nitrogen (FN) minimal medium containing 0.4% fructose, Fructose–Glycerol–Nitrogen (FGN) medium with 0.2% fructose and 0.2% glycerol, or Glycerol–Fructose–Nitrogen (GFN) containing 0.4% glycerol and 0.05% fructose, or Glycerol–Nitrogen (GN) 0.4% glycerol. The basic FN consisted of 100 mL 10 × H16 buffer, 850 mL water, and the following sterilized solutions: 10 mL NH_4_Cl (20% w/v), 1 mL MgSO_4_ × 7H_2_O (20% w/v), 1 mL CaCl_2_ × 2H_2_O (1% w/v), 1 mL FeCl_3_ × 6H_2_O (0.5% w/v), 1 mL NiCl_2_ (0.0% w/v), and 10 mL fructose (40% w/v). The 10X H16 buffer contained 90 g Na_2_HPO_4_ × 12 H_2_O and 15 g KH_2_PO_4_ ad 1 L H_2_O (final pH of 7.0). Bacteria were cultivated aerobically on a shaker at 120 rpm and 30 °C. Pre-cultures for inoculation were grown under the same conditions, but at 37 °C [3, 14, 15, 31].

Aerobic and micro-aerobic (oxygen-limited) conditions for cultivation experiments were conducted using 1000 mL baffled flasks with either 220 mL/330 mL FGN (48 h) or 800 mL GFN (80% GFN) (168 h) [10, 31], or 800 mL BSGH (72 h) (80% BSGH). 1.5% of bacterial pre-cultures were added to the growth medium which was about 10^5^ of colony-forming units of bacterial cells.

BSG were kindly supplied by Prof Frank-Jürgen Methner, (Department of Brewing Science, Technical University of Berlin, Berlin, Germany) and the “Kilikia” beer factory (Yerevan, Armenia). BSG was pre-treated by using dilute acid methods in a steam sterilizer for 1 h, 121 °C [23, 24] to obtain BSGH. The pH was adjusted to pH 7.0 using KOH.

### Bacterial growth characteristics: determination of cell size parameters by computational analysis

Bacterial biomass was determined using a Spectro UV–VIS Auto spectrophotometer (Labomed, Los Angeles CA, USA). The specific growth rate of the bacteria, µ, was determined as lg2/doubling time [20, 24, 32]. CDW of bacteria was measured to estimate bacterial biomass yield and expressed in g L^−1^, as described [32].

Computational analysis of *R. eutropha* H16 was performed using the grain analysis method for which images taken with a digital camera JAME (Japan) connected with light microscope were used; these images were analyzed by NOVA and LabView computer programs [33]. The settings of the NOVA software allowed the removal of particles smaller than 0.2 μm and bigger than 10 μm, representing a range equivalent to the diameter range of bacteria. Grain analysis mode composed of a source image, a section of the source image, a table of geometrical parameters of the bacterial cell, such us area, average size, perimeter, and length. For the three-dimensional parameter determination, the bacterial cells were treated as cylinders with two hemispherical caps, and the volume was then calculated based on the two-dimensional parameters obtained by image analysis. The shape of bacteria (α) as a main parameter of size, was calculated by the equation: α = S/P^2^; where S is the surface area of one bacterial cell and P-the perimeter (i.e., 2D projection of bacterial cell perimeter in photo document) of that cell [33]. This parameter allowed individual bacterial cells’ physical appearance (morphology) and changes in appearance to be determined among the clusters of bacterial cells in the images of the cell suspensions.

A pH electrode was used to measure the medium pH with a HJ1131B pH-meter (Hanna Instruments, Portugal). Regulation of pH has been done using solutions of 0.1 M NaOH or 0.1 N HCl.

### Oxidation–reduction potential (ORP) determination

Bacterial culture medium ORP was measured using a pair of glass redox electrodes: platinum (Pt) (EPB-1, Measuring Instruments Enterprise, Gomel, Belarus, or PT42BNC, Hanna Instruments, Portugal) and titanium-sili-cate (Ti–Si) (EO-02, Measuring Instruments Enterprise, Gomel, Belarus) [24, 30, 32]. Pt electrode is sensitive to O_2_ and H_2_ in the medium, whereas Ti–Si electrode measures the overall ORP and is not affected by the presence of O_2_ or H_2_. Before performing the assays both electrode readings were tested in control solution, which was a combination of 0.049 M potassium ferricyanide (K_3_[Fe(CN)_6_]) and 0.05 M potassium ferrocyanide (K_4_[Fe(CN)_6_]·3H_2_O) (pH 6.86). The readings of Pt and Ti–Si electrodes in the solution at 25 °C were + 245 ± 10 mV.

### Preparation of membrane vesicles, determination of ATPase activity and intracellular pH

Membrane vesicles were isolated from bacteria by the osmotic lysis method, as described [28, 34]. Membrane vesicles were incubated with 0.2 mM DCCD for 10 min. All assays were done at 30 °C. ATPase activity was calculated by determining the amount of inorganic phosphate (P_i_) produced during the reaction of membrane vesicles with 5 mM ATP (pH 7.0) in the assay mixture (50 mM Tris–HCl buffer containing 1 mM MgSO_4_, pH 7.0) [34]. The ATPase activity was expressed in nM P_i_/(min µg protein). P_i_ was measured spectrophotometrically (Labomed, Los Angeles, CA, USA), as described [34]. The intracellular pH ([pH]_in_) was measured by the quenching of fluorescence of 9-aminoacridine (9-AA), as described [35, 36], using a Cary Eclipse spectrofluorimeter (Varian, USA) with excitation at 390 nm and emission at 460 nm. The accumulation of 9-AA by the bacterial cells was determined from the disappearance of 9-AA from the assay media. To study the effects of carbonyl cyanide m-chlorophenylhydrazone (CCCP) on cells, the reagent was added at the final concentration of 2 μM and incubation was carried out for 10 min.

### Preparation of cells extracts, isolation of MBH and determination of hydrogenase activity

*Ralstonia eutropha* HF649 strain, synthesizing *Strep*-tagged MBH, was grown heterotrophically in GFN medium supplemented with 10 µg tetracycline per mL of culture, and cells were harvested after 7 days, when an OD_436_ of 11 was reached [31].

Biomass formation of *R. eutropha* H16 was followed by measuring the optical density at 436 nm (OD_436_). When the OD_436_ reached 11–12, cells were harvested by centrifugation (6000 rpm, 4 °C for 20 min). The resulting cell pellets were frozen in liquid N_2_ and stored at − 80 °C [31].

The MBH and SH activity measurements of cell extracts grown under different conditions were studied. To prepare the cell extract, a ratio of cell wet weight and the resuspension in 50 mM K–PO_4_ buffer (pH 7.0) of 1:3 was used. One tablet per 50 mL of complete EDTA-free protease inhibitor cocktail and a small amount DNase I (final concentration of 20 μg mL^−1^) were added. The cells in the resulting suspension were disrupted by 3 passages through a chilled French pressure cell (G. Heinemann Ultraschall-und Labortechnik, Schwabisch Gmund, Germany) at 18,000 psi (124.11 MPa) or 16,000 psi (110.316 MPa). Cell debris and membranes were separated from the soluble protein fraction by ultracentrifugation (36,000×*g* at 4 °C for 45 min) (Beckman Coulter, Type 45 Ti Rotor). The soluble protein fraction in the supernatant was used immediately for SH activity measurements. The MBH-containing membrane pellet was frozen in liquid N_2_ and stored at − 80 °C.

*H*_*2*_-*oxidizing activity of MBH and total activity of whole cells* was quantified by monitoring H_2_-dependent methylene blue reduction at 570 nm and 30 °C with a Cary 50 UV–vis spectrophotometer. Aliquots of cell extracts (5 to 10 μL) or bacterial whole cells, were added to anaerobic cuvettes containing 1.9 mL reaction mixture [50 mM H_2_-saturated K–PO_4_ buffer (pH 7.0)] and methylene blue as the artificial electron acceptor [3, 14, 31]. Whole cells were harvested by centrifugation (6000 rpm, 4 °C for 20 min) and washed with K–PO_4_ buffer.

*The hydrogen*-*oxidizing activity of SH* was measured in anaerobic cuvettes with NAD^+^ as the electron acceptor at 365 nm and 30 °C with a Cary 50 UV–vis spectrophotometer. Aliquots (5 to 10 μL) of SH-containing soluble protein fraction were added to 1.9 mL reaction mixture containing 50 mM H_2_-saturated Tris/HCl buffer (pH 8.0) and 1 mM NAD^+^. One unit of hydrogenase activity was defined as the amount of enzyme that catalyzes the conversion of 1 μmol of H_2_ per min and mg of protein [3, 14, 31].

*Protein concentration* was determined according to the BCA (bicinchoninic acid) method using the Pierce^®^ BCA Protein Assay Kit (Thermo Fisher Scientific, USA) and bovine serum albumin, as the [3, 14, 31].

### Data processing and reagents used in the research

Microsoft Excel 2016 was used for data processing. The data show values determined from three independent measurements; standard average of the mean data with standard errors were determined using the corresponding Microsoft Excel 2016 function, and Student criteria (P) were considered to approve the difference in average data among different series of measurements [20, 24]. The difference was valid when P < 0.05.

Fructose, glycerol, DTT, K_3_[Fe(CN)_6_] (Carl Roth GmbH, Germany) and all other reagents used in the study were of analytical grade.

## Results

### *R. eutropha* H16 growth and ORP kinetics upon utilization of different carbon sources

**Fig. 1:**
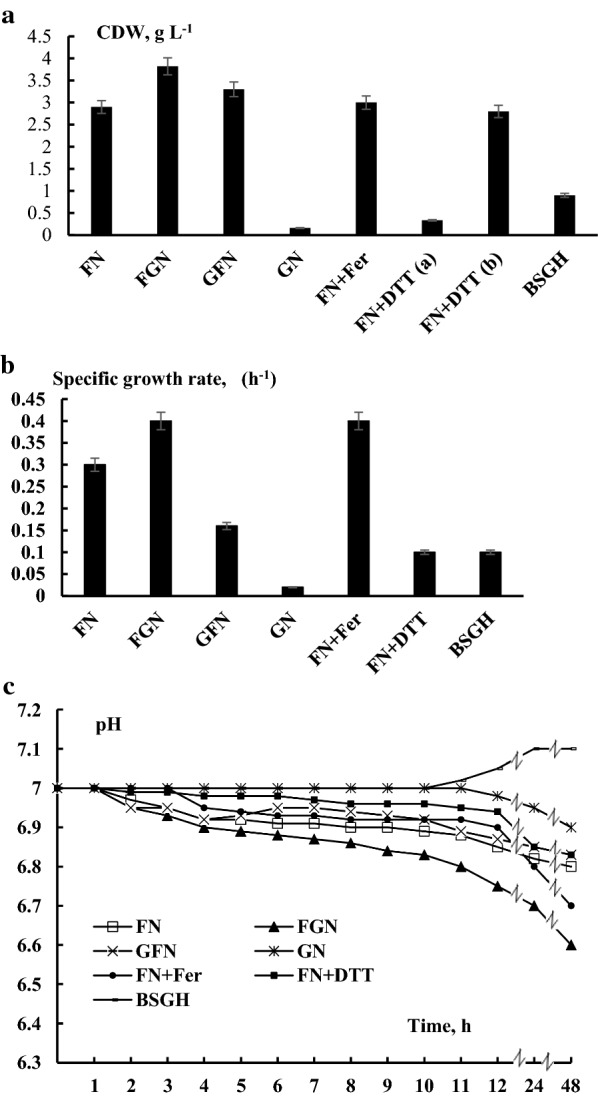
*R. eutropha* H16 growth under different conditions. The initial growth medium pH was 7.0. **a** Biomass formation, g L^−1^, was determined after 48 h bacterial growth; **b** specific growth rate (µ); **c** pH changes during bacterial growth for 48 h; *FN* Fructose–Nitrogen, *FGN* Fructose–Glycerol–Nitrogen, *GN* Glycerol–Nitrogen, *GFN* Glycerol–Fructose–Nitrogen, *BSGH* brewery spent grains hydrolysate. 3 mM DTT (DL-dithiothreitol) and 1 mM ferricyanide (Fer) were supplemented as indicated. Average data of three independent measurements are presented with standard errors

Lignocellulose (20–45% cellulose, 16% and 37% hemicellulose and 12–26% lignin) is recalcitrant towards microbial degradation [22, 23] and required therefore a pre-treatment with dilute acid, which was performed as described in “Materials and methods” to obtain BSGH. First, we grew *R. eutropha* H16 for 48 h (GFN was grown for 168 h) at 30 °C in mineral medium containing different carbon and energy sources (Fig. 1). With fructose [0.4% (w/v) we observed a biomass formation of 2.80 ± 0.02 g dry weight per L of culture (Fig. 1a)]. In the presence of 0.4% (w/v) glycerol, however, growth was negligible, revealing only 0.16 ± 0.03 g L^−1^. When growing on both substrates (0.2% fructose and 0.2% glycerol) the growth yield increased slightly by 1.2-fold (P < 0.002) to 3.4 ± 0.05 g L^−1^ (Fig. 1a). Growth on BSGH (2.5-diluted) resulted in a ~ 3.5-fold (P < 0.01) lower biomass formation when compared to the fructose-grown culture. Differences in the growth yields were reflected by the corresponding growth rates. The μ value was maximal (0.40 ± 0.01 h^−1^) for the fructose-glycerol-grown culture (Fig. 1b). A slight acidification of the culture medium was observed upon growth on fructose and/or glycerol supplementation (Fig. 1c). The largest decrease of ~ 0.4 and 0.5 pH units was observed after growth on fructose/glycerol (FGN) and glycerol/fructose (GFN, grown for 168 h). In contrast, growth of *R. eutropha* H16 on BSGH led to no noticeable change in pH (Fig. 1c).

**Fig. 2:**
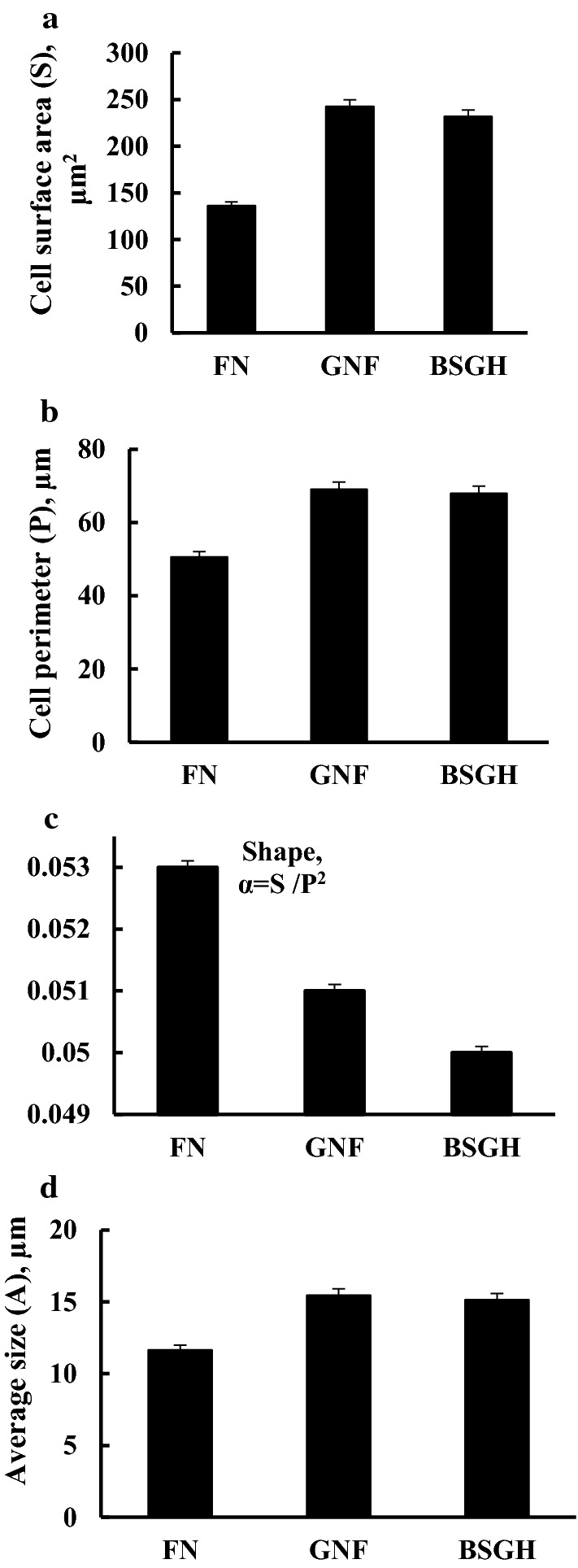
Parameters of *R. eutropha* H16 cell size under different conditions. **a** Cell surface area; **b** cell perimeter (2D projection in microscopy image); **c** shape; **d** average size. For details, see “Materials and methods” and Fig. 1

Several morphological parameters (surface area-S, perimeter-P, shape (α) average size-A) of *R. eutropha* H16 bacteria were examined after growing in different media (Fig. 2) (see “Materials and methods”): bacterial samples were considered for light microscopy after growing for 72 h in FN and in BSGH and 168 h in GNF media. The obtained results indicated changes in the bacterial cell perimeter and surface area (Fig. 2a,b), which led to the alterations in bacterial shape (α) (Fig. 2c). Bacteria have different mechanisms for maintaining cell morphology, however, in stressful (energy-limited) environments (for example BSGH and GNF), they modify the regulated process and consequently their morphology [33].

**Fig. 3:**
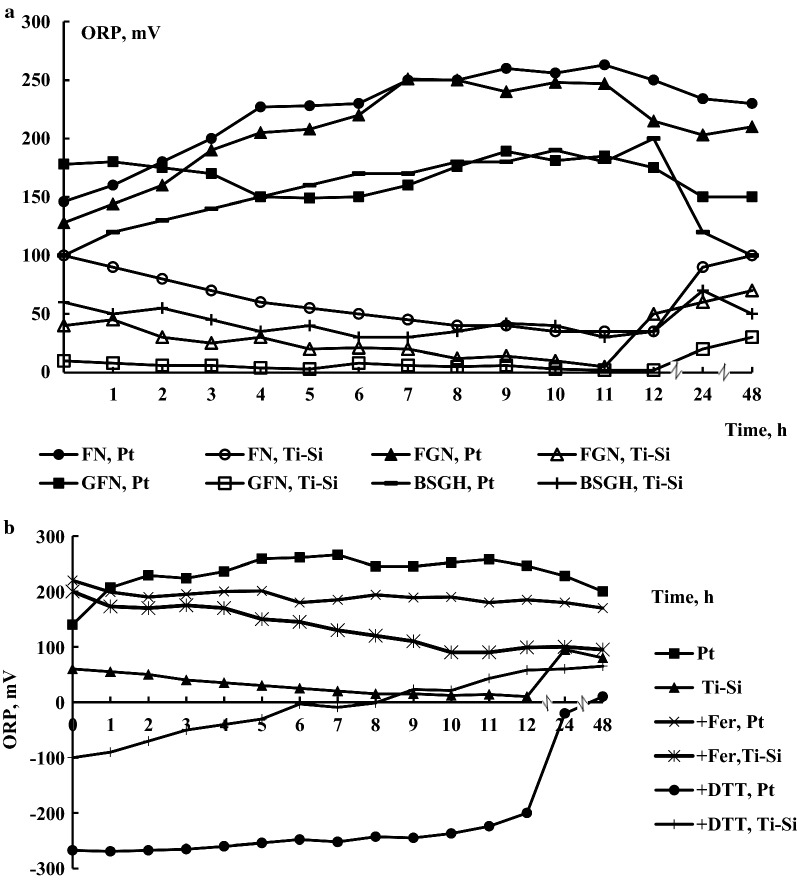
The changes of ORP by *R. eutropha* H16. **a** Kinetics under different conditions; **b** kinetics under different redox conditions. The ORP measured using Pt and Ti–Si electrodes was expressed in mV [vs Ag/AgCl (saturated by KCl)]. For the others, see the legends to Fig. 1

Figure 3a displays the changes of ORP, which was measured with two different redox electrodes as described in “Materials and methods”. The initial ORP values were + 100 ± 10 mV and + 150 ± 10 mV, measured with the Ti–Si and Pt electrodes, respectively. The behavior of the electrode readings was unusual because upon bacterial growth on both fructose and glycerol an increase for the Pt electrode reading was observed, which reached + 250 ± 10 mV during the exponential phase of growth (Fig. 3a). Moreover, it remained unaffected at 48 h of bacterial growth (see Fig. 3a). As the Pt electrode is sensitive to O_2_, this suggests that the increase in the value of the Pt electrode could be due to culture aeration. In contrast, the Ti–Si electrode reading decreased by 50 ± 10 mV during exponential growth phase. Moreover, compared to growth on BSGH, FGN, and GFN the ORP readings (Ti–Si and Pt) of bacteria grown on FN were more positive (see Fig. 3a). The ORP of the bacterial culture might be described as the relation of oxidized to reduced compounds and pH by the equation (E_h_ = E_0_ + (RT/nF) ln([ox]/[red]) + (RT/nF) ln[H^+^]) (E_h_ is ORP, E_0_ is a standard ORP, R, T and F are constants, [ox] and [red] are the amounts of oxidized and reduced compounds, respectively), following ORP decrease with increasing pH [24, 30, 32]. However, redox processes during bacterial growth are complex, and ORP decrease of the bacterial culture could not be described by the equation. Thus, the relationship of ORP decline with a reduction in pH during bacterial growth is difficult to interpret. On the other hand, the value of ORP might have effects on Hyd and other membrane-associated enzyme activities [24, 30, 32]. The change of reading of ORP in the medium of GFN (0.05% fructose and 0.4% glycerol) at 5 h was slight and might be regarded due to secretion of end products of utilization of fructose followed by use of glycerol.

### The effects of redox reagents on *R. eutropha* H16 growth and ORP kinetics

An impermeable oxidizer K_3_[Fe(CN)_6_] and membrane-permeating reducer DL-dithiothreitol were used for the application of an initial positive (~ + 200 mV) and negative (~ − 250 mV) ORP value, respectively (Fig. 3b). The reducing conditions of the growth medium adjusted with DTT, prolonged the bacterial lag phase and inhibited the growth of the bacterium for 24 h in FN (see Fig. 1a), but after 48 h the growth was restored reaching up to 2.80 ± 0.02 g L^−1^ biomass. Oxidizer K_3_[Fe(CN)_6_] both at 1 and 2 mM concentrations slightly increased (~ 1.1-fold) (P < 0.05) bacterial biomass formation (see Fig. 1a). Similarly, DTT inhibited μ, but the latter was stimulated ~ 1.3-fold (P < 0.05) upon K_3_[Fe(CN)_6_] addition (see Fig. 1b). Again, growth medium acidification (0.2 unit of pH) was observed upon both K_3_[Fe(CN)_6_] and DTT supplementation (see Fig. 1c).

Both redox (Pt and Ti–Si) electrode readings were 200 ± 10 mV upon K_3_[Fe(CN)_6_] supplementation, but again, the Ti–Si electrode reading decreased (see Fig. 3b). When the cells were grown in the presence of DTT both electrode readings changed from negative to positive values (see Fig. 3b).

### F_O_F_1_-ATPase activity of *R. eutropha* H16 membrane vesicles and intracellular pH

**Fig. 4:**
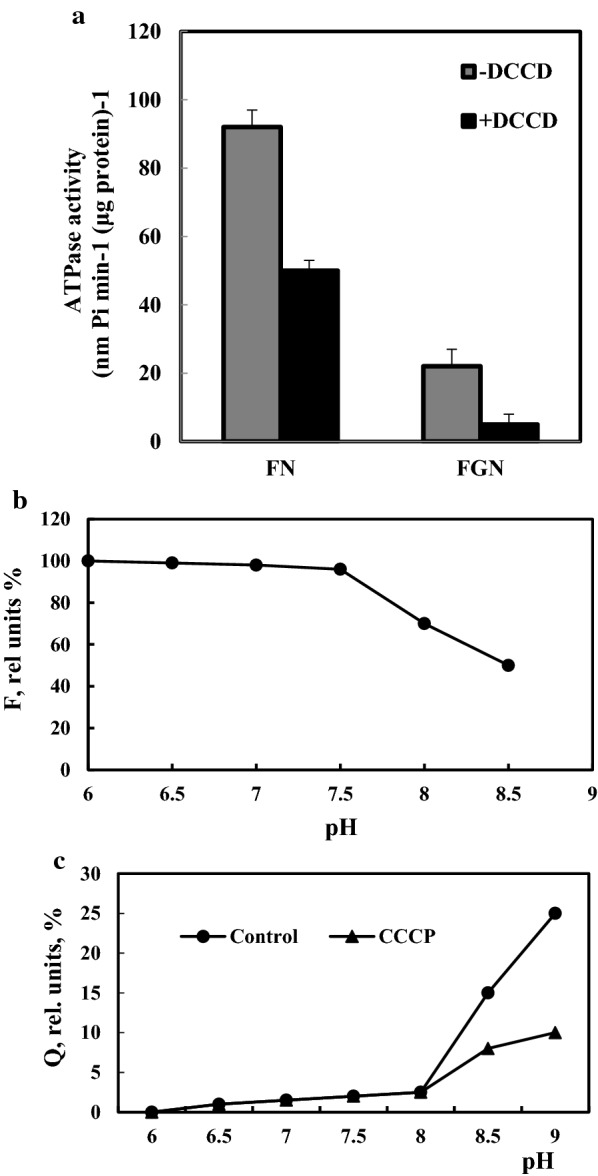
ATPase activity of membrane vesicles **a** of *R. eutropha* H16 and pH dependence of 9-AA fluorescence; **b** and its quenching; **c** induced *R. eutropha* H16. Bacteria were grown heterotrophically in minimal salts FN or FGN media. The DCCD (0.2 mM) was added into the reaction medium where indicated. The assay of pH was the same as growth pH. The fluorescence intensity (F) in relative units at pH 6.0 was taken as 100%, and the fluorescence quenching (Q) was expressed in % of the initial value of F. Addition of 2 μM CCCP eliminated the quenching. For other details, see “Materials and methods”

Bioenergetic properties of bacteria, such as changes in F_O_F_1_-ATPase activity and [pH]_in_, were investigated upon bacterial growth on glycerol and fructose. The overall ATPase activity of right-side-out membrane vesicles of *R. eutropha* H16 grown heterotrophically on FN or FGN was investigated. To determine the F_O_F_1_-ATPase activity, membrane vesicles were treated with the inhibitor (DCCD) for 10 min (see “Materials and methods”). The ATPase activity upon utilization of only fructose (FN medium) was 92 ± 5 nMol Pi (min μg protein)^−1^ and 0.2 mM DCCD inhibited ATPase activity ~ 2.3-fold (P < 0.01) (Fig. 4). It is worth noting that, compared to growth in FN, membrane vesicles demonstrated ~ 4.2- (P < 0.002) and ~ 2.5-fold (P < 0.05) lower F_O_F_1_-ATPase activity upon fructose and glycerol co-utilization (FGN medium), respectively (Fig. 4a).

The distribution of 9-AA between extra- and intracellular spaces in the bacterial cells reflects the pH gradient across the cytoplasmic membrane. Dependence of 9-AA fluorescence on the medium pH without bacteria is presented in Fig. 4b: the intensity of fluorescence remained constant and decreased insignificantly at pH below 7.5. The fluorescence quenching by addition of *R. eutropha* H16 occurred when [pH]_out_ was higher than [pH]_in_ (Fig. 4c). The [pH]_in_ measured by the 9-AA quenching was 8.00 ± 0.05. As a control experiment, 9AA fluorescence quenching was investigated upon protonophore supplementation: The fluorescence quenching was eliminated by supplementation of 2 μM CCCP (see Fig. 4c).

### Hydrogenase activity of *R. eutropha* H16 utilizing organic waste materials

**Fig. 5:**
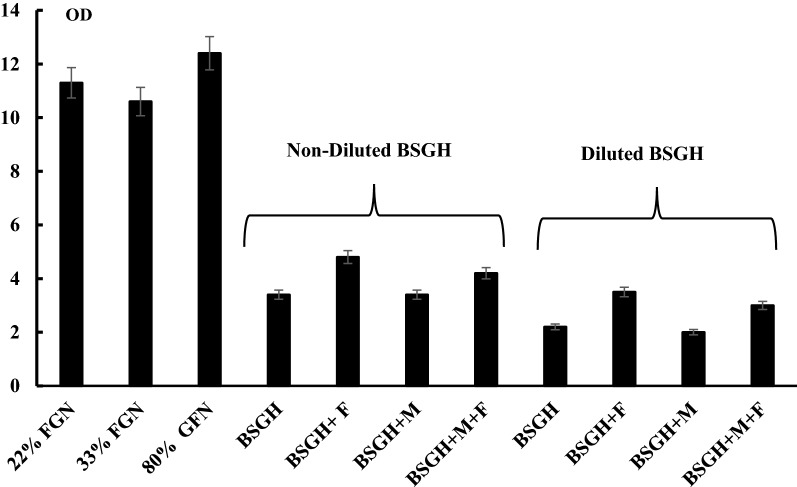
Growth of *R. eutropha* H16 under different conditions. FN-Fructose—F—0.05% fructose, M–Ni and Fe ions. For the other details, see the legend to Fig. 1

**Fig. 6:**
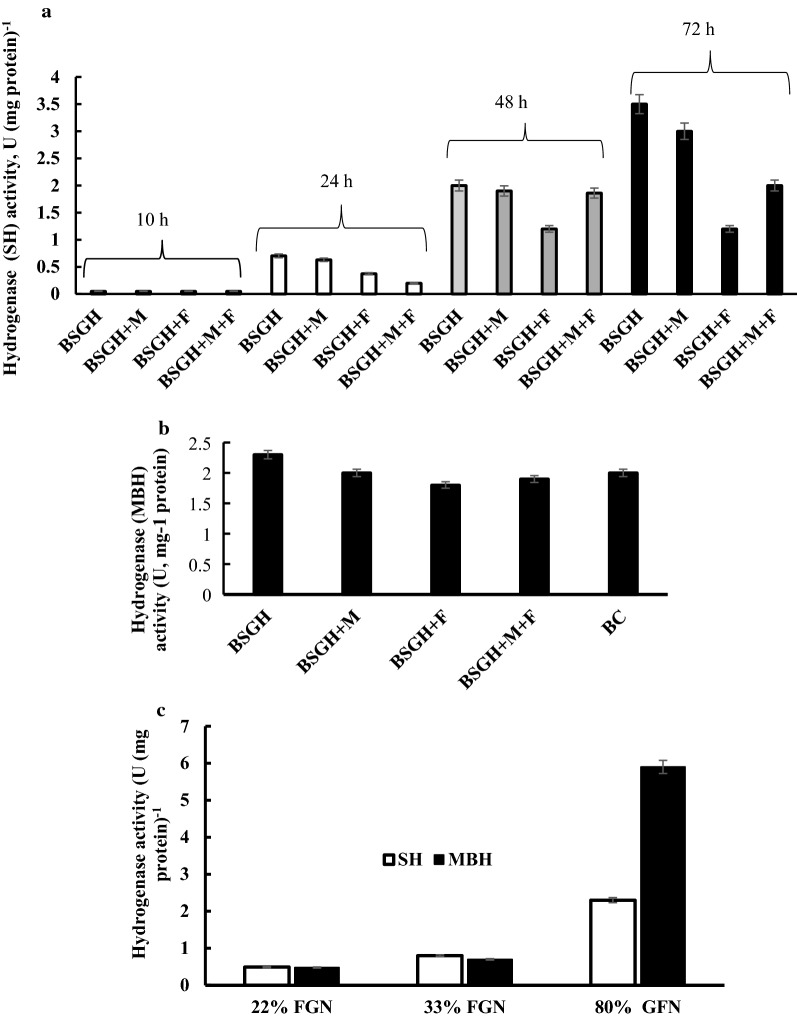
Hyd activity of *R. eutropha* H16 bacterial cell extracts during bacterial growth under different conditions. F—0.05% fructose, M–Ni and Fe ions, *BC* buffering components. **a** SH activity on BSGH; **b** MBH activity on BSGH; **c** MBH and SH activity after growth in GFN medium. One unit of Hyd activity was defined as the amount of enzyme that catalyzes the conversion of 1 μmol of H_2_ per min. For the other details, see “Materials and methods” and the legend to Fig. 1

*Ralstonia eutropha* H16 was cultivated under oxygen limiting conditions with different carbon sources in 1000-mL flasks filled to 22% (FGN_22_) and 33% (FGN_33_) of the total volume with FGN or to 80% with GFN (see Methods). In the case of FGN, an OD_436nm_ of ca. 11 was reached after 48 h of growth, whereas in GFN the same amount of biomass was formed after ca. 168 h (7 days) of growth (Fig. 5). The cells were harvested, and cell extracts were prepared, in which the MBH and SH activities were determined. In soluble extracts of cells grown in FGN_22_ and FGN_33_, the SH activity was 0.50 ± 0.01 and 0.80 ± 0.02 U mg^−1^, respectively (Fig. 6c). The SH activity was stimulated ~ 3-fold (P < 0.002) higher in extracts of GFN-grown cells. Consistently, in the case of MBH activity, membrane extracts of GFN-grown cells showed ~ ninefold (P < 0.002) higher activity (up to 6.00 ± 0.03 U mg^−1^) than extracts from cells grown in FGN_22_ and FGN_33_ (see Fig. 6c). MBH was purified when the maximal enzyme activity was detected in the late stationary growth phase after 7 days: the activity of the purified MBH was ~ 140 U mg^−1^. Electrophoretic analysis showed that we obtained purified protein (data not shown). The results are in accordance with the findings obtained previously, where under energy limitation (limited oxygen and fructose) conditions, upon bacterial growth on glycerol the Hyd enzyme synthesis and activity are increased [3, 14].

Several modifications of BSGH medium (e.g. non- or 2.5-fold dilutions using distilled water), or supplementing with 0.05% of fructose, or trace element solutions (Ni^2+^, Fe^3+^, Ca^2+^, Mg^2+^ ions) or buffer (Na–K salts) were investigated to achieve the highest cell growth and SH and MBH activity. Moreover, biomass formation (OD_436_) and SH and MBH activities were also estimated during bacterial growth for 10, 24, 48 and 72 h: as Ni^2+^ and Fe^3+^ ions are necessary for Hyd enzyme biosynthesis [1], influence of these ions on Hyd activity and growth of bacteria was also studied. Growth in non-diluted BSGH resulted in a ~ 1.5-fold (P < 0.002) higher biomass formation than in diluted BSGH. Moreover, supplementation of non-diluted BSGH with 0.05% fructose stimulated biomass formation by  ~ 1.5-fold (P < 0.002) (final OD_436_ of ~ 5) (see Fig. 5). As expected, the addition of Ni and Fe ions had no effect on bacterial growth.

SH and MBH activities were investigated under the described growth conditions: maximal SH activity (3.50 ± 0.02 U mg^−1^) was observed in extracts from cells grown in non-diluted BSGH without any supplementation. Despite 0.05% fructose stimulating bacterial growth, the SH activity was decreased by ~ threefold (P < 0.001) (Fig. 6a). The addition of Ni and Fe ions (1 mM) had no, or even a negative, effect on SH and MBH activities.

Consistently, MBH activity was highest in membrane extracts of cells grown on non-diluted BSGH. Notably, it was ~ 1.5-fold (P < 0.002) higher than in cells grown in FGN_33_, but ~ fourfold (P < 0.002) lower than in cells grown in GFN (Fig. 6a, b). So far, all growth experiments with BSGH medium were done in 1000-mL flasks filled with just 200 mL medium under air. Total Hyd activity was also studied under O_2_-limiting conditions, with 800 mL BSGH medium in 1000-mL flasks and is discussed below.

It should be noted, that the experiments described above relied on the self-buffering capacity of BSGH medium. However, the addition of Na/K-based buffer components to BSGH medium had no significant effect on bacterial growth and Hyd activity (Fig. 6b). Hyd activity was examined during bacterial growth for 10, 24, 48 and 72 h in non-diluted BSGH without or with Ni, Fe and fructose supplementation (Fig. 6a). There was no significant Hyd activity after bacterial growth of 10 h (OD_436_ was ~ 1.3), whereas, after 24 h (OD_436_ was ~ 3) 0.7 ± 0.01 U (mg protein)^−1^ SH activity was observed. At 48 h of growth SH activity was ~ 2±0.01 U (mg protein)^−1^, which reached its maximum of 3.5 ± 0.02 U (mg protein)^−1^ after 72 h of growth (OD_436_ was ~ 4) (Fig. 6a).

Thus, maximal Hyd activity was detected for *R. eutropha* H16 after 72 h of growth, late stationary growth phase when grown on BSGH. More importantly, similar activities for SH and MBH were obtained upon bacterial growth on BSGH obtained from “Kilikia” beer factory (Yerevan, Armenia), indicating that growth and hydrogenase activity were unaffected by different sources of BSGH.

### H_2_-oxidizing total hydrogenase activity of whole cells of *R. eutropha* H16 under different conditions

**Fig. 7:**
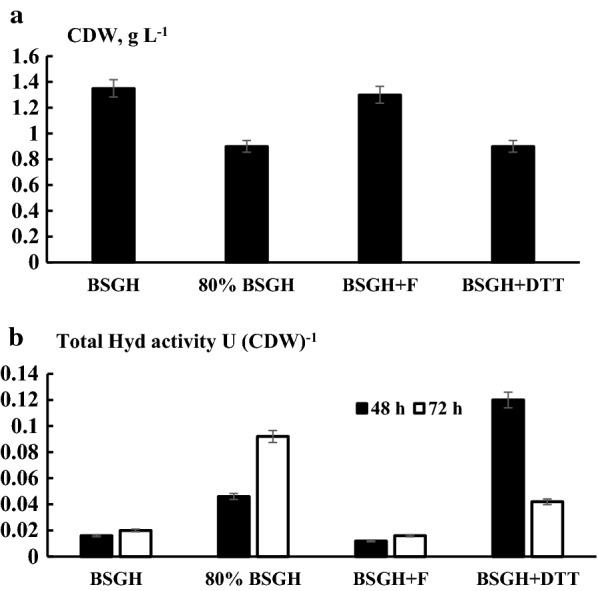
Bacterial growth (**a**) and total Hyd activity of *R. eutropha* H16 whole cells (**b**) under different conditions. 3 mM DTT (DL-dithiothreitol) and 1 mM ferricyanide (F) was supplemented where indicated. For the other details, see the legend to Fig. 1

Due to the existence of different Hyds having various roles, it is of significance to evaluate the total Hyd activity of cells, because whole cells might have different applications. To observe the overall effects of experimental conditions (mainly, micro-aerobic conditions and presence of redox reagents) on Hyds, total H_2_-oxidizing activity was investigated in bacterial whole cells. Moreover, this approach might offer insight into how growth on BSGH affects Hyd enzyme synthesis. Total H_2_-oxidizing Hyd activity of whole cells of *R. eutropha* H16 was measured after 48 h and 72 h of growth in BSGH in the presence of different supplements by monitoring H_2_-dependent methylene blue reduction at 570 nm. The results were compared with the data of BSGH without additional supplementation. Compared to growth and standard conditions in BSGH, oxygen limitation (micro-aerobic conditions, 80% BSGH) led to a ~ 1.5-fold (P < 0.001) decrease in CDW (Fig. 7a). However, the total Hyd activity was stimulated by ~ fivefold (P < 0.001) after 72 h of growth (Fig. 7b). It is worth noting that total Hyd activity of cells grown in GFN (7 days) and FGN (2 days) was close to values of BSGH and threefold (P < 0.002) lower than values of BSGH.

The effects of the redox-regulating compounds DTT (3 mM) and K_3_[Fe(CN)_6_] (1 mM) were also investigated. The presence of 3 mM DTT decreased bacterial growth (see Fig. 7a), and K_3_[Fe(CN)_6_] had no effect, which is in line with the corresponding observations for growth in FN (Figs. 1, 3). K_3_[Fe(CN)_6_] had also no effect on Hyd activity, but, interestingly, DTT stimulated Hyd activity ~ eightfold (P < 0.001) after 48 h of growth (Fig. 7b). The stimulating effect of DTT was less pronounced after 72 h of growth.

Moreover, negative values of ORP (readings of Ti–Si and Pt electrodes) up to 100 ± 0.10 mV were observed upon bacterial growth in BSGH under micro-aerobic conditions.

## Discussion

In this study, the focus was directed to SH and MBH of *R. eutropha* H16, which are promising candidate enzymes for H_2_-based biofuel application. As was mentioned above, heterotrophic growth can be supported by a variety of organic substrates, but fructose and glycerol are commonly used with this bacterial species. It was stated that under heterotrophic conditions using a defined FGN medium a substrate shift occurs from the preferentially utilized carbon source (fructose), to the less-preferred glycerol resulting in de-repression of the *hox* regulon, which harbors Hyd structural and auxiliary genes [3, 12, 14]. Note, in FGN and GNF conditions 0.5- and 8-fold less fructose, respectively, are supplemented into the growth medium, which might also lead to more economical biomass and formation of valuable products.

SH and MBH activities were observed upon bacterial growth on BSGH, FGN and GFN. Our results have shown that additional supplementations (fructose, microelements) have no or a negative influence on MBH and SH activities. Thus, bacterial biomass and active Hyds can be easily obtained with less expense and efforts using only BSGH. It seems BSGH has sufficient buffering capacity and all necessary microelements for *R. eutropha* H16 growth [21, 25, 26]. However, pretreatment of lignocellulosic biomass often involves side reactions resulting in lignocellulose-derived by-products that are inhibitory to bacterial metabolism [22, 26]. Thus, compared to FN and FGN, less biomass formation was observed upon bacterial growth of *R. eutropha* H16 on BSGH. The differences in these morphological parameters of the bacteria shown in this study are probably due to the bacterial stress response [33]. Yet, BSG can be considered as a useful new source of bacterial biomass and Hyd enzyme production, particularly because fructose and other components of the FN or GFN medium are much more expensive substrates than BSG [21, 24, 37]. A significant amount of BSG is derived annually from breweries. Therefore, providing wet BSG with a low price to local buyers, like farmers (for use as cattle feed, etc.) or biogas stations will be a good solution for the breweries to remove the BSG, since this is a cheap alternative that does not require the energy expenditure to dry BSG. However, transportation costs of BSG are expensive and should be considered [24, 37]. Moreover, it has been observed that different BSG sources [BSGs from Department of Brewing Science, TUB (Berlin, Germany), and “Kilikia” beer factory (Yerevan, Armenia)] similarly affect bacterial growth and Hyd activity. Total hydrogenase activity of whole cells was also investigated under different conditions: microaerobic conditions promoted Hyd activity in BSGH. Moreover, supplementation of DTT significantly stimulated Hyd activity.

With the pH decrease a concomitant decline in the ORP reading of the Ti–Si electrode was observed (Fig. 1c, 2). The ORP decrease might be due to secretion of redox-active metabolites into the medium, accordingly lowering exterior pH [27, 28]. Nevertheless, changes in redox properties of the proteins of the bacterial cell surface might result in ORP disturbance [27, 29]. Moreover, compared to FN, the ORP values were more reducing upon bacterial growth on BSGH and FGN and GFN. Thus, upon growth on FGN or BSGH the relationship of Hyd enzyme derepression with low (more reductive) ORP is suggested. Future studies are still required to understand redox regulation of Hyd enzyme biosynthesis. It is suggested that ORP can be controlled by supplying oxidants and reductants or can be mediated by redox stress. As an example, non-penetrating oxidizers suppress *Escherichia coli* growth and decrease acidification of the medium by inducing positive ORP values [30]. On the other hand, reducing conditions of the environment adjusted with DTT, lead to increased formation of formic acid by *E. coli* [27]. Therefore, culture medium ORP may affect the yields of produced metabolites. Consequently, ORP could be considered as a tool to monitor growth and changes in metabolism of aerobically growing bacteria to optimize yields of end metabolites.

In the case of *R. eutropha* H16 more oxidizing conditions of the environment induced by K_3_[Fe(CN)_6_] stimulated bacterial growth. In contrast, reducing (negative values achieved by DTT) conditions suppressed bacterial growth. DTT is an unstable compound, which can react with oxygen during bacterial growth, but yet growth suppression of bacteria was noted for first 24 h of growth. Nevertheless, bacteria were able to overcome redox stress after 48 h, and the growth was recovered and most importantly total Hyd activity was stimulated in BSGH.

It was also stated that, reducing conditions induced by DTT stimulated the F_O_F_1_-ATPase activity in *Geobacillus toebii* pointing to a role for the F_O_F_1_-ATP synthase in redox sensing by this bacterium [28]. In addition, the relationship between F_O_F_1_-ATP synthase and Hyd enzymes during fermentation of different carbon sources has been documented [20]. The FoF_1_ATPase activity of membrane vesicles of *R. eutropha* H16 was investigated and in contrast to FN the overall ATPase- and FoF_1_ ATPase-activities of membrane vesicles of bacteria grown on FGN were significantly decreased. Low ATPase activity upon bacterial growth on FGN might be an indicator of energy limitation and subsequently lead to Hyd gene derepression.

Thus, MBH and SH are attractive targets for potential application in biochemical hydrogen fuel cells. *R. eutropha* H16 can grow upon cheap carbon sources and bacterial growth and SH and MBH activities were observed during degradation of lignocellulosic BSGH. Redox regulation of bacterial growth was investigated and oxidizing conditions stimulated bacterial specific growth rate. In contrast, reducing conditions suppressed bacterial growth, but Hyd activity was stimulated. It is interesting that a decrease in ORP during growth of the bacteria was observed, which was more significant upon growth on BSGH and FGN. Relationship of ORP with Hyd enzyme activity might be suggested by these data. Further studies are required to reveal the mechanisms of redox stress adaptation by *R. eutropha* H16 and Hyd activity, where the main role might be contributed by FoF_1_-ATPase.

## Conclusions

High biomass formation of the non-pathogenic *R. eutropha* H16 can be easily achieved. However, high production costs for bacterial large-scale production of biomass and biotechnologically valuable products are still an economic challenge. The application of inexpensive raw materials could significantly reduce costs. Moreover, it has been demonstrated that energy-limitation favors the catabolic derepression of Hyd gene expression [3, 14]. This feature allows isolating biomass with active Hyd, avoiding an autotrophic fermentation process, which requires the use of dangerous gas mixtures of H_2_, O_2_ and CO_2_. These new studies identify the optimal conditions for *R*. *eutropha* H16 bacterial growth and associated Hyd activity on BSGH. In this context, consumption of cheap waste products (BSG and glycerol) will provide low-priced bacterial biomass and Hyd enzymes formation.

## Data Availability

All data generated or analyzed during this study are included in this published article.
